# Infralimbic Cortex Biases Preference Decision Making for Offspring over Competing Cocaine-Associated Stimuli in New Mother Rats

**DOI:** 10.1523/ENEURO.0460-19.2020

**Published:** 2020-07-13

**Authors:** Mariana Pereira, Joan I. Morrell

**Affiliations:** 1 Department of Psychological and Brain Sciences, University of Massachusetts, Amherst, MA 01003,; 2 Center for Molecular and Behavioral Neuroscience, Rutgers, The State University of New Jersey, Newark, NJ 07102

**Keywords:** addiction, conditioned place preference, maternal behavior, medial prefrontal cortex, postpartum period

## Abstract

In the context of drug abuse, converging evidence suggests that cocaine use in new mothers is significantly reduced by the competing motivation related to child rearing. Given the demonstrated importance of the medial prefrontal cortex (mPFC) in decision-making processes, we investigated the contribution of distinct regions of the mPFC [anterior cingulate (Cg1), prelimbic (PrL), infralimbic (IL)] to decision making in new mother rats performing a concurrent pup/cocaine choice conditioned place preference (CPP) task. When given a choice, inactivation of IL cortex significantly biased decision making of mother rats toward cocaine-associated cues, highly contrasting the distribution of preferences by control groups. In contrast, inactivation of PrL cortex had the opposite effect, significantly increasing offspring bias in the decision making, such that none of the mothers chose the cocaine-associated alternative. Cg1 inactivation was without effect. Functional inactivation of these same mPFC subregions had no effect in a non-conflict CPP task in which context-induced cocaine or pup seeking were examined separately. Notably, inactivation of the IL cortex also interfered with maternal behavior. Taken together, we have identified a specific role of the IL cortex in the prioritization of offspring over drug competing alternatives, thus promoting resistance to drug use in new mothers.

## Abstract (precis)

In the context of drug abuse, converging evidence suggests that cocaine use in new mothers is significantly reduced by the competing motivation related to child rearing.

## Significance Statement

Cocaine use by postpartum women is a serious health problem that has a tragic impact on the mother’s ability to properly care for her child, with life-long consequences for both the mother and her child. Here, we show that in the context of new motherhood, the infralimbic (IL) cortex biases decision making toward offspring stimuli over cocaine seeking in new mothers. These findings provide novel information about how the maternal brain processes information about offspring and how this information is integrated to bias decision making. Considering the major impact of maternal cocaine use on both mother and child health, understanding how maternal motivation can provide resistance to drug use is highly pertinent.

## Introduction

Cocaine use in new mothers is a serious clinical problem, often with detrimental impacts on the mother-child relationship and child development (Roland and Volpe, 1989; Burns et al., 1991; Ball et al., 1997; Forray and Foster, 2015). Notably, evidence strongly suggests that in the context of substance use, competition from motivation to care for the child can significantly reduce substance use, promote and/or support abstinence in new mothers (Richardson and Day, 1991; SAMHSA, Center for Behavioral Health Statistics and Quality, 2018). Accordingly, substance use interventions that promote the mother-infant relationship are generally more effective at achieving and/or facilitating maintenance of maternal abstinence, many for the first time (Ashley et al., 2003; McComish et al., 2003; Mullins et al., 2004; Porter and Porter, 2004; Suchman et al., 2010; Rutherford and Mayes, 2019). Although considerable work has accumulated regarding the neurobiology of cocaine seeking, very few studies have investigated the neurobiology of the interaction with parenting and the decision-making processes unique to new mothers.

Offspring are powerful motivationally relevant stimuli for new mothers, promoting significant attention and sustained caregiving (Lee et al., 2000; Pereira and Morrell, 2010). Understanding how the maternal brain processes information about offspring and how this information is integrated to bias decision making will provide crucial insights for developing intervention strategies to prevent postpartum relapse to drug use, with health benefits for both mothers and their children.

The prefrontal cortex (PFC) is critically involved in cognitive and executive functions necessary for the optimal selection of behavior. Substantial evidence from animal and human studies confirms the direct involvement of the PFC in aspects of addiction, including drug seeking, craving and relapse evoked by drugs, stress, and drug-conditioned stimuli (Ciccocioppo et al., 2001; Capriles et al., 2003; Rebec and Sun, 2005; Di Pietro et al., 2006; Moorman et al., 2015). Similarly, evidence indicate that the PFC is critically involved in cognitive and affective processes key to parenting (Afonso et al., 2007; Febo et al., 2010; Leuner and Gould, 2010). The rodent medial PFC (mPFC) is functionally homologous to the primate dorsolateral PFC and includes the anterior cingulate (Cg1), the prelimbic (PrL), and the infralimbic (IL) cortices (Heidbreder and Groenewegen, 2003; Uylings et al., 2003; Vertes, 2006). These regions have divergent patterns of connectivity to corticostriatal circuitry and subserve distinct and dissociable behavioral functions (Groenewegen et al., 1997; Ragozzino et al., 1999; Groenewegen and Uylings, 2000; Heidbreder and Groenewegen, 2003; Killcross and Coutureau, 2003; Dalley et al., 2004; Vertes, 2004; Gabbott et al., 2005; Moorman et al., 2015).

Our prior findings showed a critical involvement of the medial preoptic area (mPOA), which has important reciprocal connections with the mPFC in biasing behavioral allocation toward offspring related stimuli when challenged by a cocaine alternative (Pereira and Morrell, 2010, 2011). The present study extends these earlier findings to examine the circuitry involved in maternal prioritization of offspring by examining the specific contribution that the medial prefrontal Cg1, PrL, and IL cortices make to preference decision making between competing offspring-associated and cocaine-associated alternatives in new mothers. To this aim, these cortical subregions were transiently inactivated with bupivacaine in trained mother rats performing a concurrent pup/cocaine choice conditioned place preference (CPP) task. In the present study, preference decision making was differentially altered by inactivation of the PrL and IL cortices. Additional experiments were conducted to clarify the specific nature of the disruption in preference decision making induced by subregion-specific mPFC inactivation.

## Materials and Methods

### Animals

Primiparous postpartum Sprague Dawley female rats (original stock from Charles River Laboratories), bred at Rutgers University AAALAC-accredited Research Animal Facility, were used in all experiments. All animals were maintained in a temperature-controlled room on a 12/12 h light/dark cycle (lights on at 7:00 A.M.), with *ad libitum* access to water, rat chow (Lab Diet 5008, PMI Nutrition International, LLC), and sunflowers seeds. Before giving birth, pregnant females were housed in individual cages lined with woodchip bedding (Beta chip, Northeastern Products Corp.) and containing shredded paper towels as nest-building material. Postpartum females remained with their pups for 24 h after parturition, without interruption. On postpartum day (PPD) 1 (birth = day 0), litters were culled to four male and four female pups per mother. Behavioral testing occurred during the light phase of the light/dark cycle. All experiments followed the Society for Neuroscience’s Policies on the Use of Animals and Humans in Neuroscience Research and were approved by the Rutgers University Animal Care and Use Committee.

### Surgery

On day 1 postpartum, females were anesthetized with 1 ml/kg intraperitoneal injection of a solution containing 75.0 mg/ml ketamine HCl, 7.5 mg/ml xylazine, and 1.5 mg/ml acepromazine maleate. Rats were then secured in a stereotaxic frame (David Kopf Instruments) with the incisor bar set at −3.2 mm (flat skull). Females were bilaterally implanted with 22-gauge stainless steel guide cannulae (Plastics One) aimed at above one of the mPFC subregions: Cg1: anterior-posterior (AP) from bregma +3.0 mm, medial-lateral (ML) from the midline ±0.5 mm, and dorso-ventral from the skull surface (DV) −2.0 mm; PrL: AP +3.0 mm, ML ±0.5 mm, DV −3.0 mm; and IL: AP +3.0 mm, ML ±0.5 mm, DV −4.0 mm. Cannulae were secured to the skull with stainless steel screws and cranioplastic cement. Dummy stylets were inserted in the guide cannulae to maintain patency until infusions were made. Immediately after surgery, females were reunited with their pups in their home cages and allowed to recover before behavioral testing.

### Microinjection procedure

Two days before testing, females were gently handled once daily to habituate them to the experimenter handling associated with the intracranial drug administration. On PPD8, just before behavioral testing, females were gently hand-held while their stylets were removed and replaced by 28-gauge stainless steel injectors that extended 0.75 mm for Cg1, and 1.5 mm for PrL, and IL past the tip of the guide cannulae. Injectors were connected by PE-10 tubing to 10-μl Hamilton syringes, and bilateral infusions were driven simultaneously by a two-syringe infusion pump (Harvard 22 syringe pump; Harvard Apparatus).

Inactivation of mPFC subregions was achieved by infusion of bupivacaine hydrochloride (2% w/v solution; Sigma) as previously published (Pereira and Morrell, 2009, 2010). Bupivacaine reversibly blocks voltage-gated sodium channels and hence prevents initiation and propagation of action potentials both in neuronal cell bodies and axons (Hille, 1966). We chose bupivacaine over other pharmacological agents due to its fast onset of action (within minutes), shorter duration of action (up to 1 h), and more discrete functional spread (∼radius of 500–620 μm from the tip of the injector; Tehovnik and Sommer, 1997; Boehnke and Rasmusson, 2001; Edeline et al., 2002; Pereira de Vasconcelos et al., 2006).

**Figure 1.**
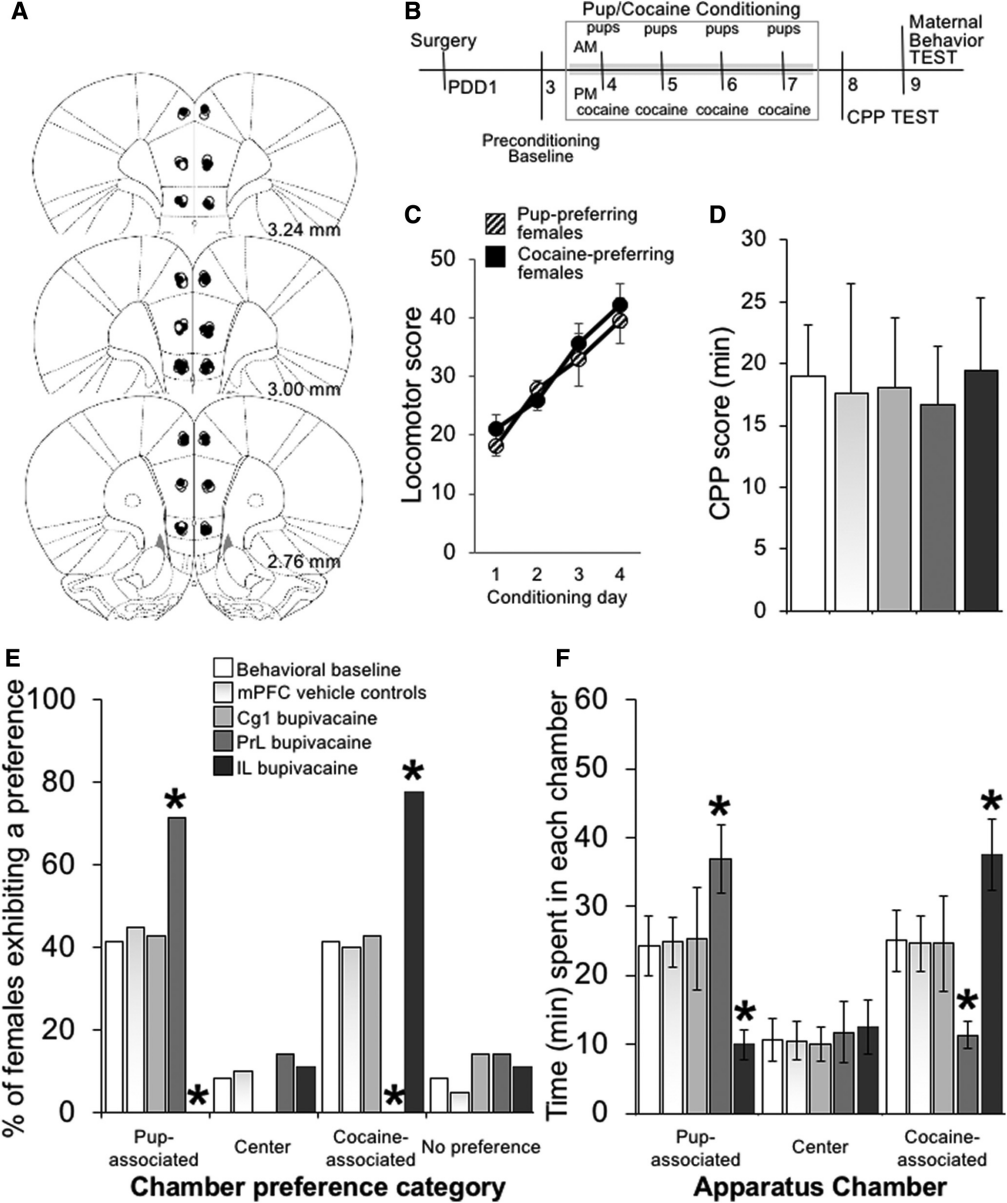
Effect of transient mPFC subregion-specific inactivation on preference decision making in a concurrent pup/cocaine choice CPP. ***A***, Schematic representation of cross-sections of the rat brain showing the location of injection sites in mother rats receiving infusions of saline (white circles) or bupivacaine (inactivation, black circles) into each of three mPFC subregions. Numbers beside each plate indicate the distance caudal to bregma in millimeters. ***B***, Experimental timeline. ***C***, Locomotor scores during 1.0 mg/kg intraperitoneal cocaine conditioning in the CPP apparatus across the four conditioning days. ***D***, CPP scores (time spent in the preferred compartment during the postconditioning test minus the time spent in the same compartment during the preconditioning session), (***E***) conditioned chamber preferences, and (***F***) mean time spent in each chamber of the CPP apparatus during the test session by behavioral control (*n* = 12), mPFC vehicle treated (*n* = 20), and mPFC Cg1 (*n* = 7), PrL (*n* = 7), and IL (*n* = 9) bupivacaine-treated postpartum females. Immediately before the start of the 60-min postconditioning CPP test, mPFC cannulated females randomly received bilateral intracranial infusions of either 2% bupivacaine or saline vehicle. Statistical analysis revealed that there was no significant difference in distribution of preference on concurrent pup/cocaine choice between postpartum females that received vehicle into Cg1, PrL, or IL subregions. As such, these groups were combined into a single vehicle-treated group for graphing purposes. Data are expressed as the mean ± SEM; *significant difference at *p* < 0.05.

**Figure 2.**
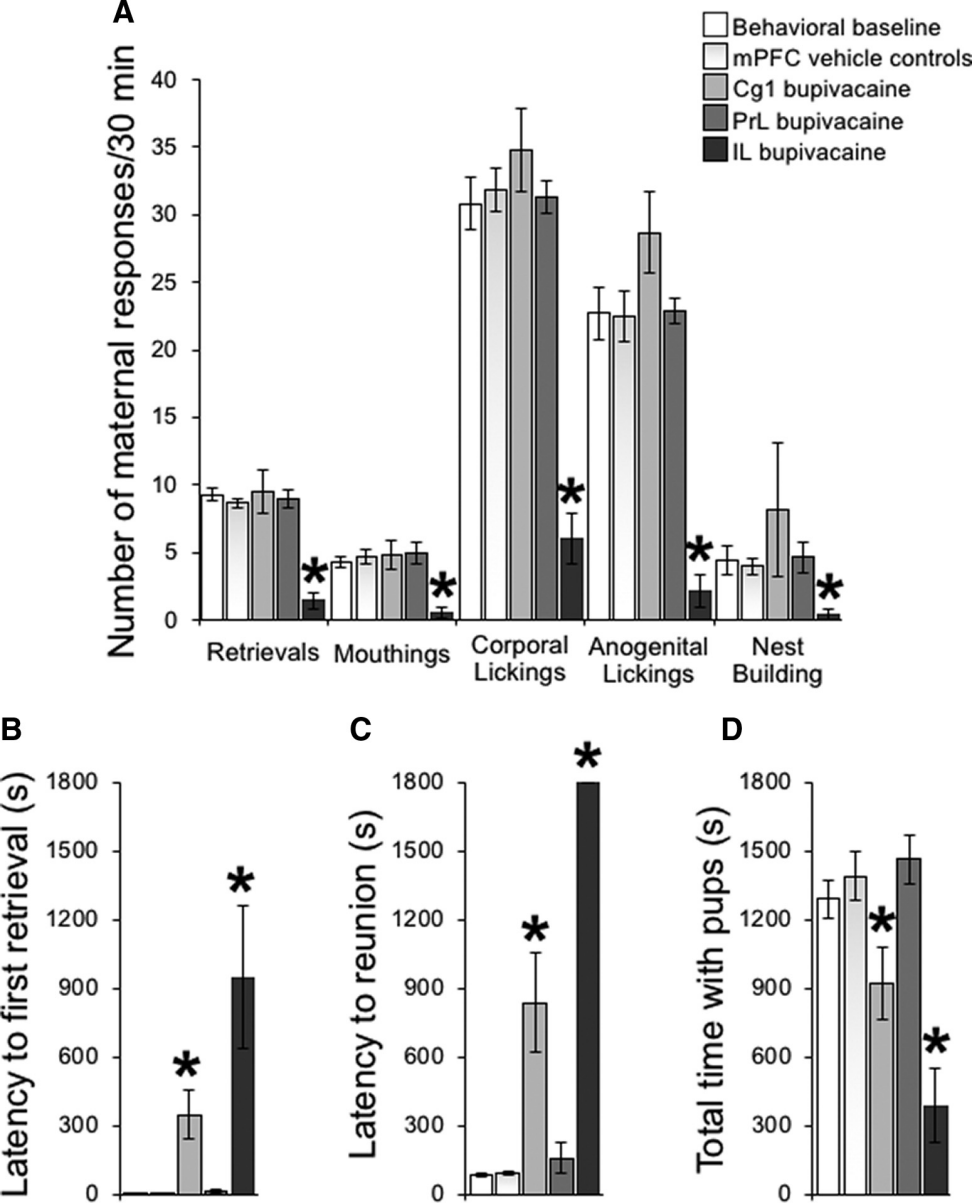
Effect of transient mPFC subregion-specific inactivation on maternal behavior. ***A***, Number of active maternal responses over the 30-min maternal behavior test following infusion of either 2% bupivacaine or saline vehicle into the Cg1, PrL, or IL. ***B***, ***C***, Latency to first retrieval and reunion of the litter, and (***D***) duration of total time with pups over the maternal behavior test. Data are expressed as the mean ± SEM; *significant difference at *p* < 0.05.

Infusions of bupivacaine or saline were administered bilaterally into one of the mPFC subregions over 120 s at a rate of 0.5 μl/min. The injectors were left in place for an additional 60 s to allow for diffusion of the drug. Immediately after, stylets were replaced. Each rat remained in its home cage for a 5-min period before behavioral testing. An infusion volume of 1.0 μl rather than a smaller 0.5-μl infusion volume was selected based on our prior results and those of others demonstrating a more stable inactivation over time while producing a similar functional spread (Tehovnik and Sommer, 1997; Pereira and Morrell, 2009). Additional groups of postpartum females that did not undergo surgery but were similarly handled, trained and tested as the surgical groups were used to provide behavioral baseline. All females remained healthy throughout the experiment, fully exhibiting typical maternal behaviors, and their pups gained weight and developed normally (Figs. 1 and 2).

### Conditioned Place Preference task

CPP training and testing was conducted as previously described (Pereira and Morrell, 2010) using a custom-made CPP apparatus with three equal-sized compartments made of clear Plexiglas and connected by manually operated guillotine doors. Each side compartment contained distinct contextual cues that remained present throughout all conditioning and testing sessions, of wallpaper and tactile flooring, becoming a unique environment for the association learning and testing of the CPP procedure. The center compartment had white wallpaper and solid gray floor. Luminance (measured with a Konica Minolta Luminance Meter LS-100) was equal in all three compartments. Subject location and activity within the CPP apparatus were recorded automatically via photograph beam breaks that are input to a computer interface with Med-PC IV Research Control and Data Acquisition System software (Med Associates Inc.).

CPP experiments consisted of a 5-d schedule with three distinct sequential phases, preconditioning baseline, conditioning training, and postconditioning testing.

#### Preconditioning baseline session

On day 4 postpartum, unconditioned (baseline) preferences were determined by allowing females complete access to the CPP apparatus for 60 min. The apparatus was balanced such that postpartum females averaged equal amounts of time spent in both conditioning side compartments during this session, and compartment assignment was counterbalanced on a group basis. Preconditioning testing revealed that the groups were balanced, such that each group averaged equal amounts of time in each conditioning side compartments.

#### Conditioning phase

Conditioning sessions were conducted twice daily for four consecutive days, from PPD4 to PPD7. Experimental groups of postpartum females were randomly assigned to and trained in one of three CPP procedures: (1) a concurrent pup/cocaine choice CPP, (2) a cocaine-induced CPP, or (3) a pup-induced CPP.

Cocaine hydrochloride (National Institute of Drug Abuse) was daily dissolved in 0.9% saline solution at a concentration of 1.0 or 5.0 mg/ml. Injection volumes (intraperitoneally) of cocaine and saline were 1 ml/kg. The cocaine-conditioning session was always last each day, ensuring at least 12 h for residual cocaine to clear from females’ circulation before subsequent conditioning sessions. No postpartum females developed any skin lesions or other pathology in the abdominal cavity or the mammary glands due to intraperitoneal cocaine injections.

**Figure 3.**
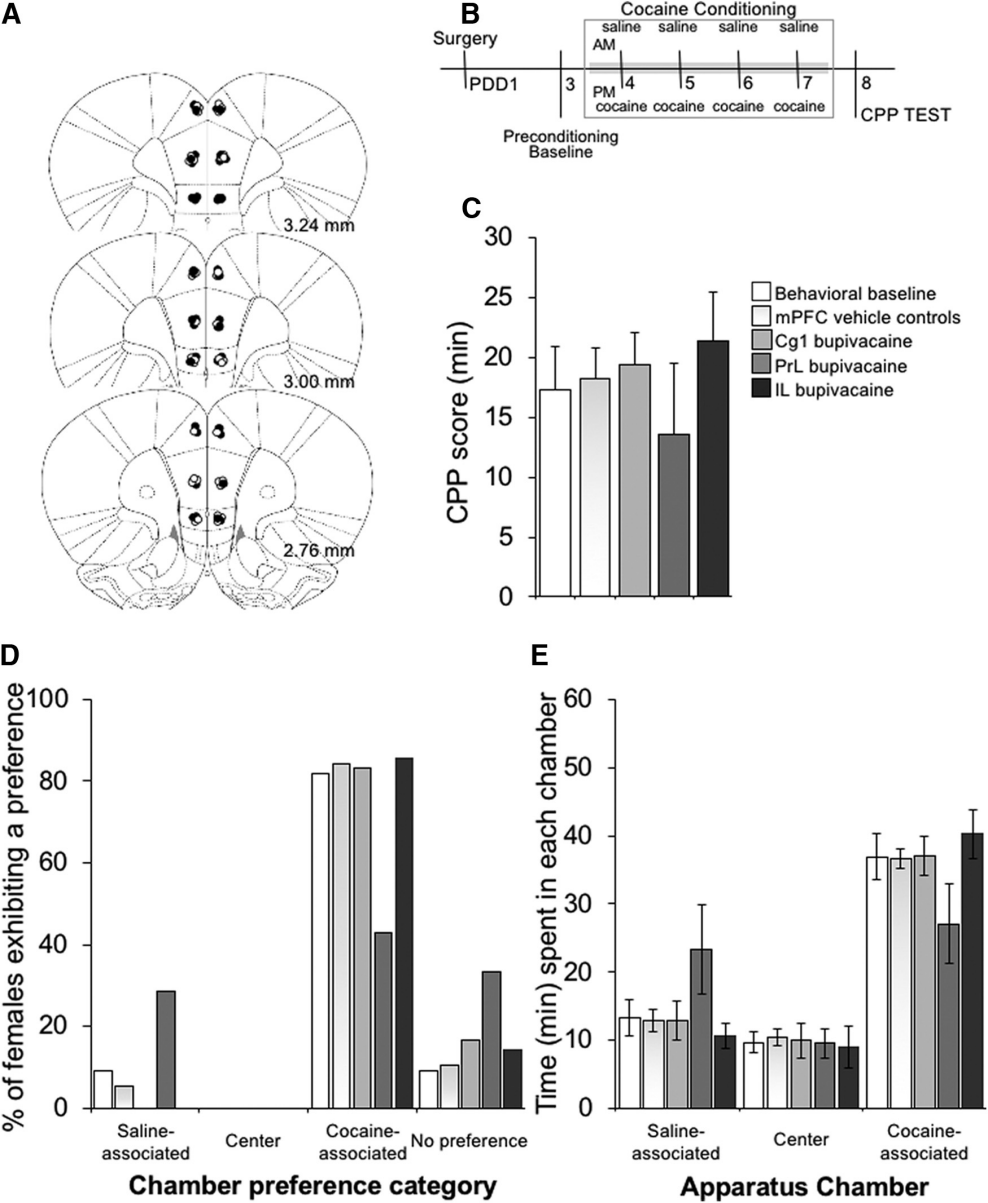
Effect of transient inactivation of the mPFC subregion-specific on expression of cocaine-induced CPP. ***A***, Schematic representation of cross-sections of the rat brain showing the location of injection sites in mother rats receiving infusions of saline (white circles) or bupivacaine (inactivation, black circles) into each of three mPFC subregions. Numbers beside each plate indicate the distance caudal to bregma in millimeters. ***B***, Experimental timeline. ***C***, CPP scores (time spent in the cocaine-associated compartment during the postconditioning test minus the time spent in the same compartment during the preconditioning session), (***D***) conditioned chamber preferences, and (***E***) mean time spent in each chamber of the CPP apparatus during the test session by behavioral control (*n* = 11), mPFC vehicle treated (*n* = 19), and mPFC Cg1 (*n* = 6), PrL (*n* = 7), and IL (*n* = 7) bupivacaine-treated postpartum females. All further details as in Figure 1. Data are expressed as the mean ± SEM; *significant difference at *p* < 0.05.

**Figure 4.**
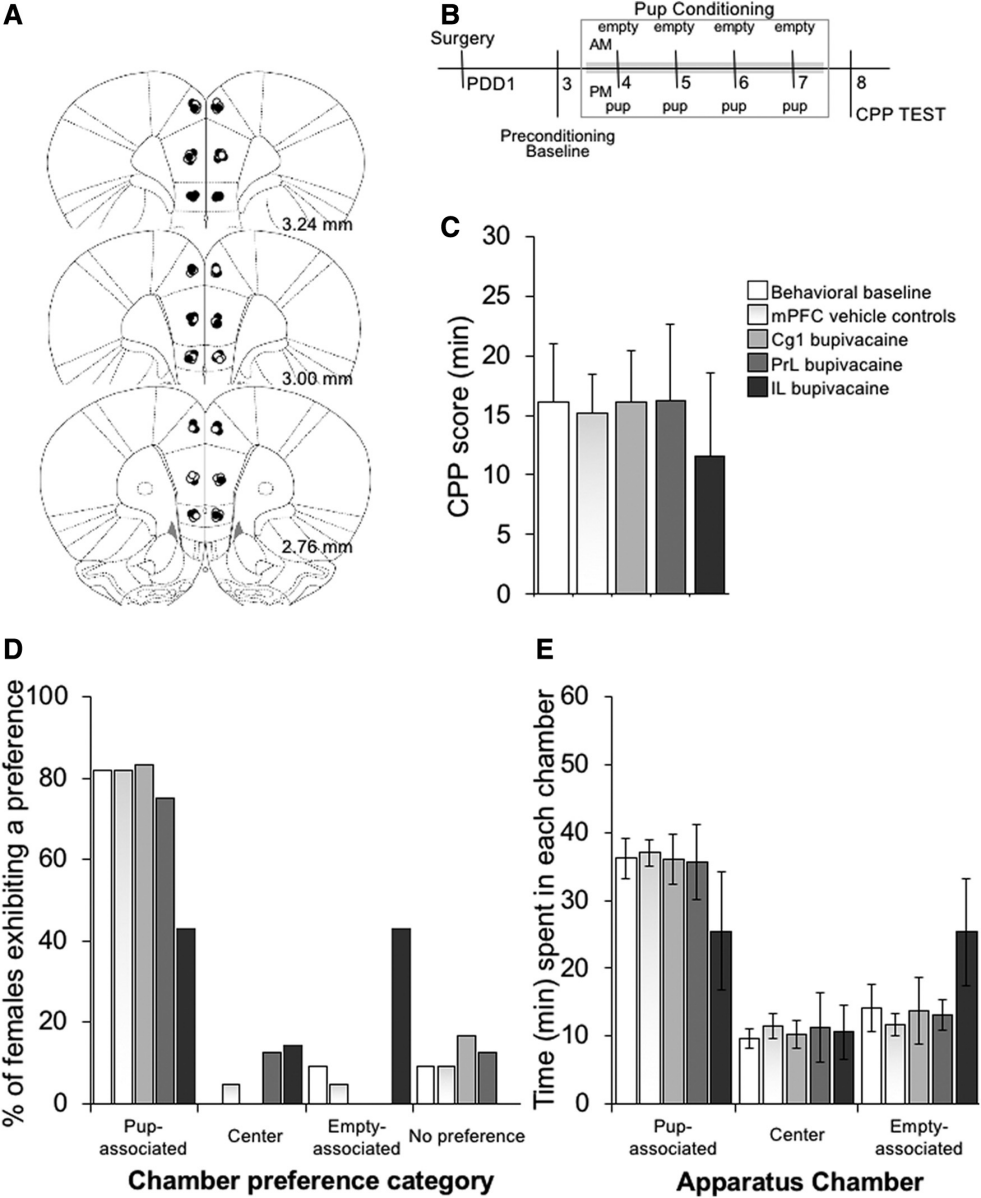
Effect of transient inactivation of the mPFC subregion-specific on expression of pup-induced CPP. ***A***, Schematic representation of cross-sections of the rat brain showing the location of injection sites in mother rats receiving infusions of saline (white circles) or bupivacaine (inactivation, black circles) into each of three mPFC subregions. Numbers beside each plate indicate the distance caudal to bregma in millimeters. ***B***, Experimental timeline. ***C***, CPP score (time spent in the pup-associated compartment during the postconditioning test minus the time spent in the same compartment during the preconditioning session), (***D***) conditioned chamber preferences, and (***E***) mean time spent in each chamber of the CPP apparatus during the test session by behavioral control (*n* = 11), mPFC vehicle treated (*n* = 22), and mPFC Cg1 (*n* = 6), PrL (*n* = 8), and IL (*n* = 7) bupivacaine-treated postpartum females. All further details as in Figure 1. Data are expressed as the mean ± SEM; *significant difference at *p* < 0.05.

For the concurrent pup/cocaine choice CPP (Fig. 1*B*), groups of females were separately conditioned to associate each side compartment with their eight-pup litter or 1.0 mg/ml/kg intraperitoneal injection of cocaine. A 1.0 mg/kg dose of cocaine was chosen for the concurrent pup/cocaine choice CPP to allow for preference categories to be represented relatively equally, and with a sufficient number of females with either a pup-associated or cocaine-associated CPP, so that the effect of a mPFC site-specific inactivation could be detected in relation to this baseline preference (Pereira and Morrell, 2010). Pup-conditioning sessions started in the morning, after females were deprived from their pups for 2 h. Litters were removed from their home cages and housed temporarily in same-litter groups in small cages. Postpartum females were then confined to the assigned pup-condition compartment for 1 h with their pups. Females (and their pups) were then returned to their home cage and left undisturbed for 2 h before receiving the cocaine-conditioning session. Females were injected with 1.0 mg/ml/kg intraperitoneal cocaine and immediately placed in the alternate conditioning compartment for 30 min. For cocaine-induced CPP (Fig. 3*B*), groups of females were separately conditioned to associate either one or the other side compartments with administration of saline or 5.0 mg/ml/kg cocaine. A 5.0 mg/kg dose of cocaine was selected for cocaine-CPP on the basis of previous research showing that it produces maximal CPP responses in postpartum females (Pereira and Morrell, 2010). Immediately before each conditioning session, postpartum females were intraperitoneally injected with cocaine or same volume of saline and confined to their respective conditioning chamber for 30 min. Females were returned to their home cages and pups after each conditioning session. Conditioning sessions started in the morning for the saline condition. For the pup-induced CPP (Fig. 4*B*), groups of postpartum females were separately conditioned to associate side compartments with their eight-pup litter or no specific unconditioned stimulus (empty compartment). Conditioning sessions started in the morning for the empty compartment condition. Litters were removed from their cages 2 h before conditioning and housed temporarily in same-litter groups in small cages. Females were then returned to their pup-less home cage after the morning conditioning session for 1 h. Immediately before the pup-conditioning session, each female’s eight-pup litter was placed in the corresponding conditioning chamber. Thus, postpartum females undergo the pup-conditioning session after being deprived of pups for 2 h, and their pups, used for conditioning, have also been deprived of maternal care for the same duration.

#### Postconditioning testing session

CPP for the stimulus-associated environment was evaluated on PPD8, the day after the final conditioning session. Before testing, females received bilateral infusions (1.0 μl/side) of either 2% bupivacaine HCl or saline vehicle into one of the mPFC subregions. Assignment to bupivacaine or vehicle treatment was counterbalanced such that within each group half of the females received bupivacaine infusions and the other half received saline. Five minutes later, postpartum females were placed in the center of the CPP apparatus and allowed complete access for 60 min, with no unconditioned stimuli present. The amount of time spent in each compartment was used to assess individual conditioned environment preferences.

Females were then returned to their home cage with their pups and were all observed behaving maternally, including nursing their litters within 1 h.

### Maternal behavior testing

To examine the role of these same cortical subregions on maternal behavior, postpartum females were tested the following day in their home cage, following the same intracranial treatment (Fig. 1*B*). On the morning following CPP testing, the maternal behavior of postpartum females was evaluated to verify that there were no remaining behavioral effects from the previous day’s treatment. Note that all females were found in a well-constructed nest with their pups. Three pups were then removed from the nest and scattered in the cage, and females were observed until each pup was retrieved back into the nest, and every 5 min thereafter for 30 min. All females retrieved all three pups to the nest within the first minute and were observed licking and nursing their litters, indicative that maternal behavior of all females was completely normal.

Maternal behavior testing was conducted that afternoon. After a 10 min separation from their litters, PPD9 females were removed from their home cage, received infusions with either saline vehicle or 2% bupivacaine HCl, and were then returned to their home cage. Five minutes later, their eight pups were scattered in the home cage opposite the nest. The number of the following maternal behaviors was recorded continuously for 30 min: retrievals of the pups into the nest, mouthings (oral repositioning of the pups into the nest), full body and anogenital lickings, and nest building. In addition, the total duration of huddling behaviors, including lying in contact with pups and hovering over the pups in the nest while actively performing other behaviors (i.e., licking of pups or self-grooming), and the nursing posture kyphosis, a quiescent upright crouching over pups were recorded. Total time in contact with pups was the summed durations of huddling plus nursing behaviors. Also, the latency to retrieve each pup and to group the entire litter into the nest as well as to begin hovering over and nursing was registered. Only those postpartum females that retrieved each of their eight pups into the nest were considered to have grouped the litter. The latency to begin hovering over or nursing the pups was the first occurrence of a bout of each behavior ≥2 min in duration. A latency of 1800 s was given for any category of behavior that was not initiated (or completed, i.e., reunion of the litter) within the 30-min observation period. Other behaviors recorded included general exploration (line crosses and rearings), self-grooming, and eating/drinking.

### Verification of injection sites

After completion of behavioral testing, females were overdosed with sodium pentobarbital (100 mg/kg, i.p.), and perfused transcardially with 0.9% saline followed by 10% formalin. Brains were removed and postfixed in 10% formalin for 48 h before being stored in a 20% sucrose solution. The brains were sliced on a cryostat at 40-μm thickness, and cross-sections were mounted on glass slides and stained with cresyl violet. Histologic analysis was performed on slides coded to ensure that their review was conducted naive to the behavioral data, and placement of the injection sites was verified with reference to the neuroanatomical atlas of Paxinos and Watson (2007). Figures 1*A*, 3*A*, 4*A* present schematic cross-sections of the rat brain showing injector tip placements within the three medial prefrontal subregions for rats included in the analyses (*n* = 146). The data from 15 rats were removed from the analysis due to inaccurate placement. Concerning the location of the implant sites, histologic analysis indicated that, in general, all groups had bilateral injections sites located between 3.24 and 2.76 mm anterior to bregma.

### Statistics

Preconditioning environment preferences and times were compared with those of postconditioning, to provide evidence of conditioning. CPP was operationally defined as a statistically significant increase in conditioned preferences of individuals and group time spent in the preferred (pup/cocaine-associated) environment postconditioning relative to preconditioning baseline. A female was categorized as having a preference for a particular apparatus environment if spent at least 50% of the total session time in one compartment and that was at least 25% greater than either time spent in any other compartment. Females that did not meet the preference criterion were categorized as having no preference. A CPP score was calculated by subtracting the time spent in the cocaine (or pup) paired environment during the postconditioning test from the time spent in this same environment during the preconditioning test. The χ^2^ goodness-of-fit test was used to analyze categorical data from preconditioning and postconditioning environment preferences of the individual females. Between-group environment preference comparisons were examined using χ^2^ test of independence and Fisher’s exact test. Environment times were analyzed using two-way repeated measures ANOVAs, with group as the between-subject variable, and compartment and session (preconditioning vs postconditioning) as within-subject factors. CPP scores were analyzed using one-way repeated measures ANOVAs with group as the between-subject variable. Locomotor scores during cocaine conditioning sessions in the concurrent pup/cocaine choice CPP were calculated by dividing the number of new beam breaks during each conditioning session by the session time. The locomotor scores of cocaine-preferring versus pup-preferring females throughout conditioning were analyzed using one-way ANOVA with conditioning day as the repeated measure. Maternal behaviors were analyzed with two-way ANOVAs with brain structure and treatment as the between-subjects factors. Significant main effects and interactions were further analyzed using Tukey’s HSD tests. SPSS statistical package version 25 (SPSS) was used for all statistical analyses. In all cases, a statistical significance level of *p* < 0.05 was used for all data.

## Results

### Selective inactivation of PrL and IL oppositely bias preference decision making in new mothers

Preconditioning data revealed no initial/preexisting bias or preference to either side compartment, and, on average, postpartum females spent similar amounts of time in both conditioning side compartments of the CPP apparatus. Importantly, before conditioning, unconditioned environment preferences (Yates’ χ^2^ = 3.675, df = 18, *p* = 0.99) and times (no significant main effects or interactions: group, *F*_(6,48)_ = 0.22 *p* = 0.97 %$\eta_{p}^{2}$ = 0.027; compartment, *F*_(1,48)_ = 0.29 *p* = 0.6 %$\eta_{p}^{2}$ = 0.006; group × compartment interaction, *F*_(6,48)_ = 0.124 *p* = 0.99 %$\eta_{p}^{2}$ = 0.015) were similar and did not differ among groups [behavioral control (*n* = 12), mPFC vehicle treated (*n*_Cg1_ = 6, *n*_PrL_ = 7, *n*_IL_ =7), and bupivacaine treated (*n*_Cg1_ = 7, *n*_PrL_ = 7, *n*_IL_ = 9)].

Figure 1 shows the effects of transient inactivation of Cg1, PrL, and IL cortices during expression of place conditioning in the concurrent pup/cocaine choice CPP task. As expected, after conditioning, the majority of control females developed a preference for environments associated with one of the two unconditioned stimuli, either cocaine or pups. Analysis of the CPP times revealed a statistically significant increase in the time spent in the preferred environment during the test day compared with the preconditioning baseline preference, indicative of conditioning (group, *F*_(6,48)_ = 0.428 *p* = 0.86 %$\eta_{p}^{2}$ = 0.051; session, *F*_(1,48)_ = 278.1 *p* < 0.01 %$\eta_{p}^{2}$ = 0.853; group × session interaction, *F*_(6,48)_ = 0.56 *p* = 0.76 %$\eta_{p}^{2}$ = 0.07). Importantly, there was no significant difference in environment test preferences (χ^2^ = 0.18, df = 3, *p* = ns), times (group, *F*_(3,28)_ = 0.89 *p* = 0.46 %$\eta_{p}^{2}$ = 0.087; compartment, *F*_(1,28)_ = 0.0 *p* = 0.99 %$\eta_{p}^{2}$ = 0.000; group × compartment, *F*_(3,28)_ = 0.007 *p* = 0.99 %$\eta_{p}^{2}$ = 0.001), or preference scores (*F*_(3,28)_ = 0.251 *p* = 0.86 %$\eta_{p}^{2}$ = 0.026) between the behavioral control and vehicle microinjection groups, demonstrating that surgery, handling, and intracranial drug administration procedures neither affected the acquisition nor the expression of conditioned preference of postpartum females (Fig. 1*D–F*).

Inactivation of the PrL just before CPP testing completely biased the preference of postpartum females for the pup-associated environment, such that a statistically significant majority of mothers preferred the pup-associated environment (71%), whereas none preferred the cocaine-associated environment (0%; Fisher’s exact test *p* < 0.05; Fig. 1*E*). The opposite effect was observed following inactivation of the IL cortex. Specifically, intra-IL infusions of bupivacaine just before CPP testing biased the preference of postpartum females for the cocaine-associated environment, such that a statistically significant majority of females preferred the cocaine-associated environment (78%), whereas none preferred the pup-associated environment (0%; Fisher’s exact test *p* < 0.05; Fig. 1*E*).

As shown in Fig. 1*F*, the analysis of time in each environment during the expression test revealed a significant group × compartment interaction effect (*F*_(5,37)_ = 2.575 *p* < 0.05 %$\eta_{p}^{2}$ = 0.258), with time spent in the pup-associated environment significantly shorter in the intra-IL bupivacaine-treated group than in the vehicle-treated control group (Tukey’s HSD test, *p* < 0.05). In addition, the time spent in the cocaine-associated environment in the intra-PrL bupivacaine-treated group was significantly shorter than the respective control group (Tukey’s HSD test, *p* < 0.05). In contrast, inactivation of Cg1 cortex had no effect on choice CPP, with environment preferences (χ^2^ = 2.108, df = 6, *p* = 0.91) and times (Tukey’s HSD test, offspring-associated environment: *p* = 0.62 and *p* = 0.88, respectively; cocaine-associated environment: *p* = 0.71 and *p* = 0.96, respectively) of Cg1 bupivacaine-treated females not different from those of Cg1 vehicle-treated and behavioral control groups (Fig. 1*F*). In addition, pup-associated and cocaine-associated environment times during the expression test were similar among control and Cg1 groups (Tukey’s HSD test, behavioral baseline *p* = 0.93; Cg1_vehicle_ *p* = 0.94; Cg1_bupivacaine_ *p* = 0.95). However, the time spent in the pup-associated or cocaine-associated environment by PrL or IL bupivacaine-treated females was significantly longer than their time spent in the cocaine-associated or pup-associated environment, respectively (Tukey’s HSD test, *p* < 0.05; Fig. 1*F*).

#### Cocaine locomotor sensitization occurred in all mothers regardless of their CPP preference

Postpartum females exhibiting a pup-associated or a cocaine-associated environment preference similarly showed a significant increase in locomotor activity in response to cocaine across conditioning sessions (group, *F*_(1,43)_ = 0.47 *p* = 0.497 %$\eta_{p}^{2}$ = 0.01; day, *F*_(3,41)_ = 21.4 *p* < 0.05 %$\eta_{p}^{2}$ = 0.61; group × day, *F*_(3,41)_ = 0.57 *p* = 0.64 %$\eta_{p}^{2}$ = 0.04), suggesting that preference for offspring-associated over cocaine-associated environments in the concurrent pup/cocaine CPP task was not the result of a lack of responsiveness in these postpartum females to the effects of cocaine (Fig. 1*C*).

#### A functional IL and Cg1, but not PrL, are necessary for early postpartum maternal behavior

As shown in Figure 2, only mothers receiving bupivacaine infusion into the IL cortex exhibited severe deficits in their maternal behavior. There was a main effect of brain structure and treatment, as well as a significant structure × treatment interaction for all active components of maternal behavior during the 30-min maternal behavior test (retrieval: structure, *F*_(2,37)_ = 21.6 *p* < 0.05 %$\eta_{p}^{2}$ = 0.54; treatment, *F*_(1,37)_ = 13.1 *p* < 0.05 %$\eta_{p}^{2}$ = 0.261; structure × treatment, *F*_(2,37)_ = 27.1 *p* < 0.05 %$\eta_{p}^{2}$ = 0.6; mouthing: structure, *F*_(2,37)_ = 5.8 *p* < 0.05 %$\eta_{p}^{2}$ = 0.24; treatment, *F*_(1,37)_ = 3.4 *p* = 0.073 %$\eta_{p}^{2}$ = 0.08; structure × treatment, *F*_(2,37)_ = 7.7 *p* < 0.05 %$\eta_{p}^{2}$ = 0.29; corporal licking: structure, *F*_(2,37)_ = 32.1 *p* < 0.05 %$\eta_{p}^{2}$ = 0.63; treatment, *F*_(1,37)_ = 41.3 *p* < 0.05 %$\eta_{p}^{2}$ = 0.53; structure × treatment, *F*_(2,37)_ = 42.2 *p* < 0.05 %$\eta_{p}^{2}$ = 0.69; anogenital licking: structure, *F*_(2,37)_ = 28.0 *p* < 0.05 %$\eta_{p}^{2}$ = 0.6; treatment *F*_(1,37)_ = 21.4 *p* < 0.05 %$\eta_{p}^{2}$ = 0.37; structure × treatment, *F*_(2,37)_ = 36.9 *p* < 0.05 %$\eta_{p}^{2}$ = 0.67). Tests of simple main effects indicated that intra-IL bupivacaine-treated mothers exhibited significantly fewer retrievals, mouthings, corporal and anogenital lickings, and nest buildings compare to those receiving saline infusions (all *p*s < 0.05; Fig. 2*A*). In fact, during IL inactivation, none of the seven postpartum females completed retrieving and grouping the pups into the nest; three of seven did not retrieve any pups, and the remaining four retrieved less than or equal to three pups of their eight-pup litter. In contrast, all seven females retrieved and grouped all pups into the nest following infusion of vehicle into the IL (0/7 and 7/7, respectively; Fisher’s exact test, *p* < 0.05). Litters of bupivacaine IL-treated mothers grouped themselves into the nest within 594.5 ± 118.38 s, where they generally stayed together until the end of the behavioral test. IL-inactivated females also spent significantly less time in contact with their pups, due to an increased latency to begin hovering over the pups (both *p*s < 0.05; Fig. 2*C*,*D*). In addition, there were significant main effects of structure and treatment, as well as a structure × treatment interaction effect on the duration of hovering over and nursing (hover over, structure, *F*_(2,37)_ = 6.9 *p* < 0.05 %$\eta_{p}^{2}$ = 0.27; treatment, *F*_(1,37)_ = 9.6 *p* < 0.05 %$\eta_{p}^{2}$ = 0.21; structure × treatment, *F*_(2,37)_ = 12.6 *p* < 0.05 %$\eta_{p}^{2}$ = 0.4; nursing, *F*_(2,37)_ = 10.2 *p* < 0.05 %$\eta_{p}^{2}$ = 0.36; treatment, *F*_(1,37)_ = 14.9 *p* < 0.05 %$\eta_{p}^{2}$ = 0.29; structure × treatment, *F*_(2,37)_ = 3.8 *p* < 0.05 %$\eta_{p}^{2}$ = 0.2), with intra-IL bupivacaine-treated females spending less time in contact with their litters than after receiving saline infusions or compared with the behavioral control group (*p* < 0.05; Fig. 2*D*). Importantly, infusion of saline vehicle into IL had no significant effect on any maternal component measured (Fig. 2*A–D*). Thus, postpartum females receiving vehicle infusion into the IL exhibited full maternal behavior that was not different from the unoperated behavioral control group (all *p*s = ns).

No significant differences were found between behavioral control and PrL postpartum groups in any maternal behavior measured (all *p*s = ns; Fig. 2*A–D*). Inactivation of Cg1 affected the organizational aspects of maternal behavior, resulting in increased latencies to retrieve the first pup and to group all pups into the nest compared with Cg1 vehicle-treated group (both *p*s < 0.05; Fig. 2*B*,*C*), a behavioral effect distinct from that produced by inactivation of either IL or PrL subregions. Home cage activity, including general exploration and self-grooming, was not different between groups (data not shown).

#### mPFC subregion-specific inactivation does not impact context-induced cocaine seeking

Before conditioning, unconditioned chamber preferences (Yates’ χ^2^ = 3.31, df = 18, *p* = 0.99) and times (group, *F*_(6,43)_ = 0.29 *p* = 0.94 %$\eta_{p}^{2}$ = 0.038; compartment, *F*_(1,43)_ = 3.5 *p* = 0.17 %$\eta_{p}^{2}$ = 0.075; group × compartment, *F*_(6,43)_ = 0.38 *p* = 0.89 %$\eta_{p}^{2}$ = 0.05) were similar and did not differ among groups [behavioral control (*n* = 11), mPFC vehicle treated (*n*_Cg1_ = 6, *n*_PrL_ = 7, *n*_IL_ = 6), and bupivacaine treated (*n*_Cg1_ = 6, *n*_PrL_ = 7, *n*_IL_ = 7)]. Following conditioning, a significant CPP to 5.0 mg/kg intraperitoneal cocaine occurred in all groups, with CPP preference times differing statistically between the preconditioning and postconditioning sessions (group, *F*_(6,43)_ = 0.61 *p* = 0.72 %$\eta_{p}^{2}$ = 0.08; session, *F*_(1,43)_ = 106.3 *p* < 0.05 %$\eta_{p}^{2}$ = 0.712; group × session, *F*_(6,43)_ = 0.531 *p* = 0.78 %$\eta_{p}^{2}$ = 0.07; Fig. 3*C*).

Transient Cg1, PrL, or IL inactivation before testing had no impact on the motivational responsivity of postpartum females to cocaine-related stimuli (Fig. 3*D*,*E*). Thus, there was no significant difference in chamber preference (Yates’ χ^2^ = 3.643, df = 18, *p* = 0.99) nor times (group, *F*_(6,43)_ = 0.17 *p* = 0.98 %$\eta_{p}^{2}$ = 0.023; compartment, *F*_(1,43)_ = 71.6 *p* < 0.05 %$\eta_{p}^{2}$ = 0.625; group × compartment, *F*_(6,43)_ = 1.312 *p* = 0.27 %$\eta_{p}^{2}$ = 0.15; Fig. 3*D*,*E*) between groups during the CPP test with the vast majority of postpartum females, regardless of treatment, preferring the cocaine-associated environment (Fig. 3*D*,*E*).

#### mPFC subregion-specific inactivation does not impact context-induced pup seeking

Before conditioning, unconditioned chamber preferences (Yates’ χ^2^ = 2.32, df = 18, *p* = 0.99) and times (group, *F*_(6,47)_ = 0.36 *p* = 0.9 %$\eta_{p}^{2}$ = 0.044; compartment, *F*_(1,47)_ = 1.19 *p* = 0.28 %$\eta_{p}^{2}$ = 0.025; group × compartment interaction, *F*_(6,47)_ = 0.51 *p* = 0.8 %$\eta_{p}^{2}$ = 0.061) were similar and did not differ among groups [behavioral control (*n* = 11), mPFC vehicle treated (*n*_Cg1_ = 6, *n*_PrL_ = 9, *n*_IL_ = 7), and mPFC bupivacaine (*n*_Cg1_ = 6, *n*_PrL_ = 8, *n*_IL_ = 7)]. Following conditioning, a robust CPP for the pup-associated environment occurred in all groups, with CPP preference times differing statistically between the preconditioning and postconditioning sessions (group, *F*_(6,47)_ = 0.53 *p* = 0.78 %$\eta_{p}^{2}$ = 0.063; session, *F*_(1,47)_ = 44.5 *p* < 0.05 %$\eta_{p}^{2}$ = 0.486; group × session, *F*_(6,47)_ = 0.645 *p* = 0.69 %$\eta_{p}^{2}$ = 0.076).

As shown in Figure 4*D*,*E*, the analysis of preferences and environment times during the expression test revealed no significant group differences in the expression of pup-induced CPP (Yates’ χ^2^ = 5.81, df = 18, *p* = 0.99; group, *F*_(6,47)_ = 0.247 *p* = 0.96 %$\eta_{p}^{2}$ = 0.031; compartment, *F*_(1,47)_ = 31.514 *p* < 0.05 %$\eta_{p}^{2}$ = 0.401; compartment × group, *F*_(6,47)_ = 0.84 *p* = 0.55 %$\eta_{p}^{2}$ = 0.09), with the time spent in the pup-associated environment significantly higher than the time spent in the alternative one associated with no unconditioned stimuli.

## Discussion

Our findings demonstrate that the IL and PrL cortical regions critically participate in the decision-making strategy of new mothers that resolves the conflict between competing alternatives and identify a critical role for the IL cortex in prioritizing offspring-associated over competing cocaine-associated options. Inactivation of the PrL cortex shifted CPP of mother rats toward offspring-associated cues, whereas inactivation of the IL cortex had the opposite effect, significantly biasing preference choice toward cocaine-conditioned incentives, such that almost all preferred cocaine-associated and none the offspring-associated alternative. In distinct contrast to their specific role in the choice, inactivation of the PrL or IL had no effect on CPP in the absence of conflict, indicating that neither of these cortical regions are necessary for context-induced cocaine seeking or infant seeking, respectively. Our finding regarding the lack of a role for PrL in the expression of a cocaine-CPP is consistent with some previous work (Hemby et al., 1992; although see Otis et al., 2018). Results also reveal that inactivation of the Cg1 did not alter decisions on any of the CPP tasks. In addition, the IL plays a necessary role in the regulation of early postpartum maternal behavior, since its neural inactivation severely impaired the expression of maternal behavior. Regional specificity limited to Cg1, PrL, and IL of the CPP effects was demonstrated since dissociable effects on behavior were observed when bupivacaine was infused into discrete but adjacent mPFC subregions. Importantly, the behavioral effect of bupivacaine was specific for CPP choice responding, and maternal behavior, as other behaviors, including general exploration, self-grooming, eating, and drinking were not affected by the treatment. The primary findings of the present study are discussed in detail below.

Inactivation of the PrL or IL cortices oppositely biased preference decision making of new mothers in a stimulus-specific manner and toward the competing alternative representation. Thus, even in the presence of a third neutral compartment alternative, inactivation of the PrL shifted preference toward offspring-associated environments, whereas inactivation of the IL shifted CPP toward cocaine-associated environments. These results strongly suggest that both contingencies were learned (i.e., mothers learn to differentially associate the rewarding effects of each stimuli with the environmental cues) by the time of testing and that choice was based on consideration of both contingencies, not on the learning of only one contingency. Moreover, preference for offspring-associated over cocaine-associated environments could not be explained by a reduced ability of cocaine to produce an effect, because females exhibiting an offspring-associated choice showed cocaine-induced locomotor sensitization during conditioning. Thus, those mother rats that preferred offspring-associated over cocaine-associated environments did so despite being fully responsive and sensitized to (and by) cocaine in a way similar to that of the postpartum females exhibiting a cocaine-associated preference. These data support prior findings that cocaine remains highly reinforcing in early postpartum females, inducing the most robust CPP at 1.0 and 5.0 mg/kg intraperitoneal cocaine (Seip et al., 2008; Pereira and Morrell, 2010; present results).

The fact that bupivacaine infusions altered conditioned preferences in a manner that was dependent on the specific cortical region also strongly argues against the possibility that bupivacaine-induced effects in conditioned seeking behavior were due to: (1) a nonspecific performance deficits (i.e., sensory, motor, and/or general memory impairment); (2) bupivacaine-induced novelty (i.e., a novel state produced by bupivacaine inactivation could have nonspecifically and unconditionally disrupted the expression of the CPP); or (3) induction of an aversive state. Similarly, it is unlikely that bupivacaine-induced behavioral effects were due to (4) a state-dependent effect, such that differing states between acquisition (without bupivacaine treatment) and expression (with bupivacaine treatment) may have interfered with the retrieval of the associations during testing); or (5) a discrimination impairment, such that bupivacaine inactivation of mPFC impaired the animal’s ability to discriminate between options close together in value. In addition, the lack of effect of individual mPFC subregion inactivation on pup-CPP or cocaine-CPP indicates that the observed bias in the choice cannot be attributed to a reduction in the value representation of the conditioned cues. In other words, if any of the above-mentioned possibilities accounted, for example, for why bupivacaine infusion into the IL-biased preference toward cocaine seeking, then it is unclear why any of these effects would have only selectively affected preference decision making of IL females and not of PrL and Cg1 postpartum females, and only affected responding in the choice CPP task and not in the cocaine-CPP or pup-CPP tasks.

Our findings demonstrate that IL and PrL participate in the resolution of the conflict between offspring-seeking or cocaine-seeking behavior in new mothers. This notion is in keeping with numerous behavioral studies indicating specialization of function within mPFC subregions in a wide range of decision-related processes (such as attention, conflict monitoring, and decision making) to generate context-appropriate behavior (Seamans et al., 1995; Birrell and Brown, 2000; Killcross and Coutureau, 2003; Walton et al., 2003; Dalley et al., 2004; Euston et al., 2012; Moorman and Aston-Jones, 2015). Our PrL findings are in line with a number of previous studies indicating that the PrL cortex participates in reducing conflict by biasing context-induced drug-seeking (Capriles et al., 2003; Fuchs et al., 2005; Di Pietro et al., 2006; de Visser et al., 2011; Seif et al., 2013). Similarly, it could be argued that the IL cortex also participates in the conflict resolution by inhibiting cocaine seeking (LaLumiere et al., 2012; Yousuf et al., 2019), and thus, inactivation of the IL promoted cocaine-CPP, an effect only evident in the choice-CPP and not in the cocaine-CPP due to a ceiling effect. Further, our findings suggest that the IL encodes processing of offspring-related cues. Specifically, functional inactivation of the IL, but not the PrL, cortex severely disrupted caregiving in new mothers. IL-inactivated mothers spent most of the test time away from their pups, eating, drinking, and/or resting, highly contrasting the behavior of control females who spent most of the time with their litters. This result is consistent and extend those reported earlier (Afonso et al., 2007; Febo et al., 2010), by demonstrating a specific, necessary role for the IL cortex in maternal behavior. Of note, the lack of effect of IL inactivation on pup-CPP suggests that the IL cortex does not play a direct role in processing the salience/motivational value of offspring-associated stimuli. The fact that all caregiving behaviors (both active and passive) were reduced by IL inactivation, while other similarly complex behaviors were instead facilitated, suggests that the impairments were likely specific to encoding pup stimulus priority, selection of pup-associated stimuli among multiple competing stimuli for attention (cocaine, food, water, etc), and/or action, and not due to sensory or motor deficits.

Taken together, our results indicate a fundamental role for the IL cortex in prioritizing the commitment of behavior, time, and resources toward offspring and associated stimuli and biasing decision making accordingly. In this sense, the IL cortex (as well as the primate orbitomedial PFC) has been implicated in social cognition (Amodio and Frith, 2006; Wang et al., 2011; Holmes et al., 2012; Meyer-Lindenberg and Tost, 2012; Bicks et al., 2015). An important question is how the IL cortex contributes cognitive control that allows the resolution of conflict in favor of offspring, even against powerful rewards, such as cocaine. Through its projections to the nucleus accumbens shell, amygdala, ventral tegmental area, and the mPOA (Sesack et al., 1989; McDonald, 1991; Heidbreder and Groenewegen, 2003; Vertes, 2004, 2006), the IL cortex can guarantee the high expression of caregiving behaviors during the demanding task of parenting. In particular, reciprocal connection with the mPOA (Simerly and Swanson, 1988), a site that orchestrates maternal behavior (Numan and Insel, 2003), likely further contributes to this maternal bias (i.e., amplifying the salience and significance of offspring-related stimuli). In this sense, we and others have demonstrated a role of the mPOA in maternal motivation, as the functional integrity of the mPOA is necessary to promote goal-directed behaviors for pups and the choice of pup-conditioned over competing cocaine-conditioned incentives (Lee et al., 2000; Pereira and Morrell, 2010). Future experiments should determine the phenotype and connectivity of neurons within the IL cortex that prioritize offspring-related stimuli, biasing decision making against cocaine seeking in new mothers. These findings refine our understanding on how the maternal brain processes information about offspring and cocaine, how this information is integrated to bias decision making, and have implications for the development of intervention strategies to prevent drug relapse in new mothers.

## Synthesis

Reviewing Editor: Margaret McCarthy, Univ of Maryland School of Medicine Decisions are customarily a result of the Reviewing Editor and the peer reviewers coming together and discussing their recommendations until a consensus is reached. When revisions are invited, a fact-based synthesis statement explaining their decision and outlining what is needed to prepare a revision will be listed below. The following reviewer(s) agreed to reveal their identity: Claire-Dominique Walker.

The authors have addressed my original concerns by including additional details in the methods (including justification for cocaine doses), adding the complete statistical results, and including the number of animals and effect sizes for each experiment in the results section.

A few remaining concerns:

1) The authors explain the extension of their previously published work in their rebuttal but not in their revision of the paper. In fact, they do not mention the 2011 review paper in their references. This extension of previously published data should be explicitly mentioned in the revised manuscript and reference to the 2011 review paper included. This is still a major issue with the manuscript.

2. The authors should indicate in the methods whether the investigators were blind or not to treatment groups when scoring maternal behavior.

3. Fig. 1 (con’t) should be renumbered as Fig. 2 and all following Figures renumbered.
